# Supplementary material for: Laboratory testing and on-site storage are successful at mitigating the risk of release of foot-and-mouth disease virus via production of bull semen in the USA
Source: PLoS One. 2023 Nov 7;18(11):e0294036. doi: 10.1371/journal.pone.0294036 (PMC10629637; doi:10.1371/journal.pone.0294036)
Supplement: S1 Appendix — (PDF) [file pone.0294036.s001.pdf]

S1 Appendix

Supporting information for manuscript “Laboratory testing and on-site storage are successful at mitigating the risk of release of foot-and-mouth disease virus via production of bull semen in the USA” (2023), by Anne Meyer, Jay Weiker and Rory Meyer.

Model outputs used to estimate the probability of a facility becoming infected, given that there is an FMD outbreak in the US.

**Reference**	[1]	[2]	[3]	[4]	[5]
**Model**	Adapted to US from 2001 UK model	NAADSM	USAMM-USDOS	USAMM v2.1 - USDOS	ISP version 6.0
**Scale**	Pennsylvania	Arkansas, Colorado, Kansas, Louisiana, New Mexico, Oklahoma, Texas	USA	USA	USA
**Species**	Cattle and sheep	Cattle, swine and sheep	Cattle	Cattle	Bison, cattle, goats, sheep and pigs
**Number of premises**	28,779	363,989	882,692	882,692	1,819,448
**Mean number of infected premises over outbreak**	142.9	134.2	131.6	2223.0	63.5
**Mean incidence**	0.00497	0.000369	0.000149	0.00252	0.0000349
**Notes**	Results from simulations with transmission kernel twice that for the UK	Average across control scenarios	Average across scenarios	Average across control scenarios	Worst-case scenario

Summary parameters of the distributions of intermediate variables used in the risk assessment model.

**Variable**	**Mean**	**Standard deviation**	**Median**	**95th percentile**
H	0.0025	0.0019	0.0020	0.0060
M	0.50	0.14	0.50	0.73
P_1	0.00035	0.00027	0.00028	0.00084
P_2	0.95	0.030	0.96	0.99
P_3	0.98	0.040	0.99	>0.99
P_4 (14-day storage)	0.14	0.074	0.13	0.27
P_4 (30-day storage)	0.068	0.057	0.053	0.18
P_5	0.30	0.26	0.21	0.84
P_6	0.80	0.087	0.81	0.92
P_7	0.56	0.13	0.57	0.77
P_8 (100% testing)	0.047	0.040	0.037	0.13
P_9 (14-day storage, antibody)	0.017	0.097	<0.01	0.037
P_9 (30-day storage, antibody)	<0.01	<0.01	<0.01	<0.01
P_9 (14-day storage, genome)	0.077	0.18	<0.01	0.54
P_9 (30-day storage, genome)	0.011	0.054	<0.01	0.049
S_a	0.96	0.023	0.96	>0.99
S_g	0.98	0.0082	0.98	0.99
S_m	0.87	0.072	0.87	0.99
S_p	0.95	0.040	0.96	>0.99
T_{a1} (1st antibody test)	0.82	0.27	0.95	>0.99
T_{a2} (2nd antibody test)	0.97	0.031	0.98	>0.99
T_{g1} (1st genome test)	0.25	0.24	0.14	0.76
T_{g2} (2nd genome test)	0.12	0.12	0.072	0.38

Distribution of intermediate variables used in the risk assessment model.

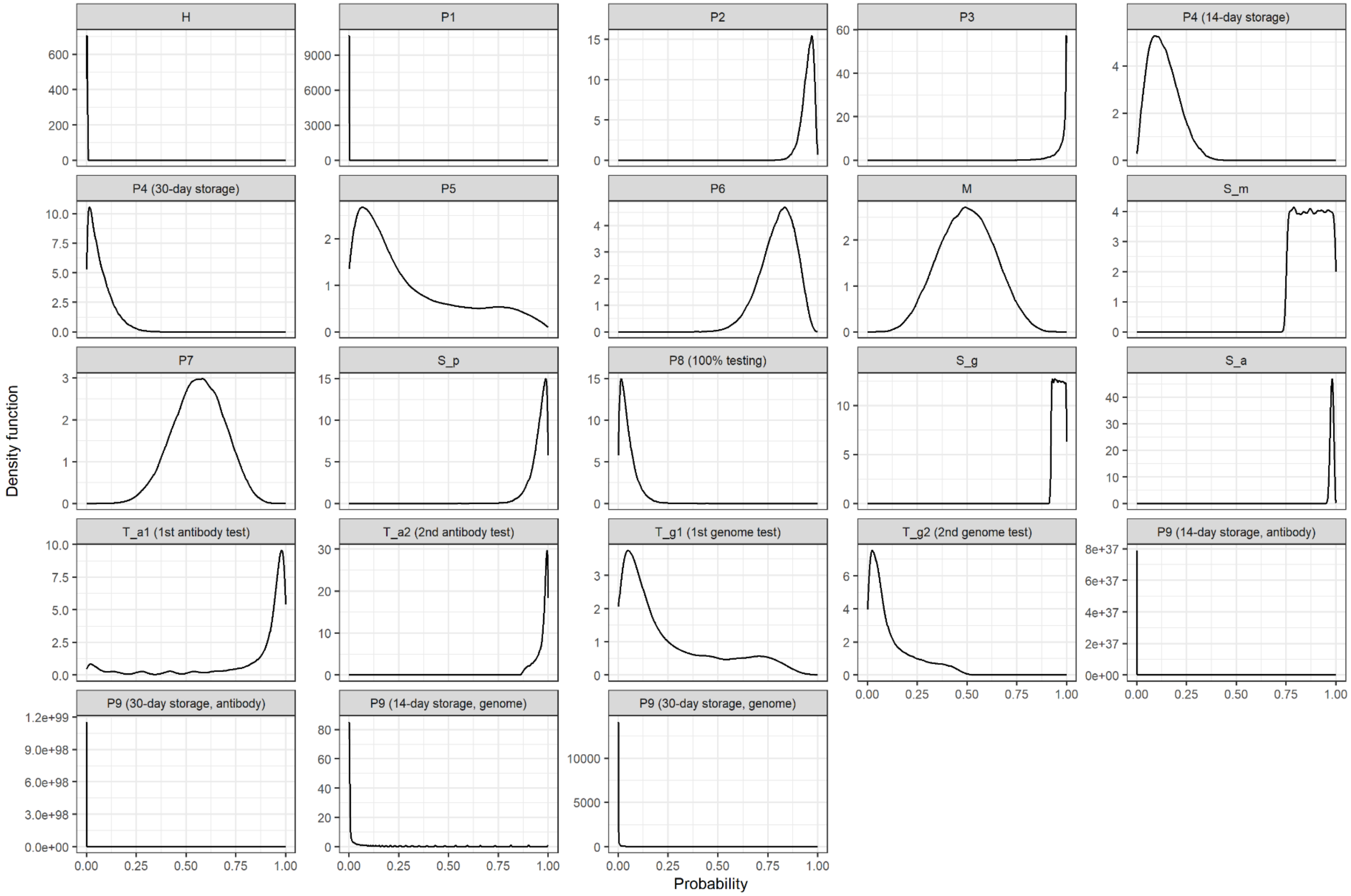

References

1. Tildesley MJ, Smith G, Keeling MJ. Modeling the spread and control of foot-and-mouth disease in Pennsylvania following its discovery and options for control. Prev Vet Med. 2012;104: 224–239. doi:10.1016/j.prevetmed.2011.11.007

2. Rawdon TG, Garner MG, Sanson RL, Stevenson MA, Cook C, Birch C, et al. Evaluating vaccination strategies to control foot-and-mouth disease: a country comparison study. Epidemiol Infect. 2018;146: 1138–1150. doi:10.1017/S0950268818001243

3. Tsao K, Sellman S, Beck-Johnson LM, Murrieta DJ, Hallman C, Lindström T, et al. Effects of regional differences and demography in modelling foot-and-mouth disease in cattle at the national scale. Interface Focus. 2020;10: 20190054. doi:10.1098/rsfs.2019.0054

4. Gilbertson K, Brommesson P, Minter A, Hallman C, Miller RS, Portacci K, et al. The Importance of Livestock Demography and Infrastructure in Driving Foot and Mouth Disease Dynamics. Life. 2022;12: 1604. doi:10.3390/life12101604

5. Yadav S, Delgado AH, Hagerman AD, Bertram MR, Moreno-Torres KI, Stenfeldt C, et al. Epidemiologic and economic considerations regarding persistently infected cattle during vaccinate-to-live strategies for control of foot-and-mouth disease in FMD-free regions. Front Vet Sci. 2022;9: 1026592. doi:10.3389/fvets.2022.1026592
